# Exploring Users’ Experiences of the Uptake and Adoption of Physical Activity Apps: Longitudinal Qualitative Study

**DOI:** 10.2196/11636

**Published:** 2019-02-08

**Authors:** Dario Baretta, Olga Perski, Patrizia Steca

**Affiliations:** 1 Department of Psychology University of Milano-Bicocca Milano Italy; 2 Department of Clinical, Educational & Health Psychology University College London London United Kingdom; 3 Institute of Digital Health University College London London United Kingdom

**Keywords:** physical activity, smartphone apps, engagement, person-based approach, longitudinal qualitative study, thematic analysis

## Abstract

**Background:**

Although smartphone apps might support physical activity (PA), engagement with them tends to be low.

**Objective:**

This study aimed to examine potential users’ needs and preferences regarding their engagement with PA apps during a first exposure to a never-used PA app and after 2 weeks’ usage.

**Methods:**

A longitudinal, one-arm qualitative study was conducted with potential PA app users. At baseline, participants (N=20) were asked to explore 1 of 3 randomly allocated PA apps while thinking aloud. Semistructured interview techniques allowed participants to elaborate on their statements. After 2 weeks, follow-up interviews explored participants’ (n=17) lived experiences of real-world app use. Verbal reports from both time points were analyzed using inductive thematic analysis.

**Results:**

Features that promote a fair and simple user experience, support users’ self-regulation skills, and address users’ exercise motives were considered important for engagement both during a first exposure and after a 2-week use of PA apps. Features that support users’ need for relatedness as well as those that facilitate users to implement their intentions were expected to be important for engagement mainly during a first exposure to PA apps. Proactive and tailored features that integrate behavioral, psychological, and contextual information to provide adaptive exercise plans and just-in-time support were considered relevant to sustain engagement over time.

**Conclusions:**

App features that address users’ exercise motives, promote self-regulation, and fulfill users’ need for relatedness might promote engagement with PA apps. Tailored and proactive features were expected to promote sustained engagement.

## Introduction

### Background

Regular physical activity (PA) is associated with a reduced risk of mortality caused by cardiovascular disease, diabetes, obesity, and some forms of cancer [1,2]. Nevertheless, worldwide, a third of adults do not meet the recommended PA guidelines [3]. Hence, the development and promotion of effective behavior change interventions that target PA is a public health priority.

A range of different behavior change interventions to increase PA have been found to be effective [4] and there is evidence supporting their cost-effectiveness [5]. However, most of these have been delivered in face-to-face settings and are hence unsuitable for targeting large, geographically diverse populations and for supporting behavior change in real time. Mobile phone technology has the potential to deliver effective PA interventions [6,7] and constitutes an economically viable tool to reach large populations [8]. Thus far, evidence suggests that Web- and mobile phone–based interventions have moderate effects on PA [8,9]; however, effect sizes are heterogeneous and have been found to vary depending on study design, mode of delivery, intervention components, outcome measures, and study length. It has, therefore, been argued that we should err on the side of caution when interpreting and generalizing early evidence from digital PA interventions [8].

It is assumed that some level of engagement with digital behavior change interventions is a precondition for their effectiveness [10]. Engagement with digital behavior change interventions has been conceptualized both as a behavior and as a subjective experience, involving dimensions of interest, attention, and affect [11].

Engagement as a behavior refers to the extent (eg, amount, frequency, and duration) of usage of a digital intervention over time. Evidence shows that digital behavior change interventions typically suffer from low usage and high abandonment rates. For example, it has been estimated that 26% of commercially available health and fitness apps are downloaded and used only once by each user and that 74% of such apps are abandoned after their tenth use [12]. The most frequently reported reasons why users abandon health apps include a lack of desired features and having abandoned one’s health goal [13]. As results from a meta-analysis of PA internet-delivered interventions found a negative relationship between early attrition and intervention effectiveness, researchers have argued that it is important to promote sustained engagement with digital PA interventions [9]. In regard to usage, a PA mobile health study has shown that although the number of user log-ins declined throughout the intervention period, it was not associated with PA behavior [14]. It has been, therefore, proposed that focusing just on broad summative usage metrics does not enable to adequately characterize and evaluate engagement with digital interventions [11,15].

Engagement as a subjective experience refers to the interaction with a digital intervention and involves feelings of interest, enjoyment, and attention [11]. By exploring this particular aspect of engagement, researchers may better understand how and why users interact with specific intervention content and design features and help explain different usage patterns [10]. For example, a review of health and fitness apps found a positive association between engagement and the inclusion of interactive features such as behavioral tracking and semi-automated options [16]. However, little attention has been paid to potential PA app users’ subjective experiences of such features. Furthermore, given that psychological factors, such as motivation, have been found or hypothesized to influence both engagement with digital behavior change interventions and the effect of specific behavior change techniques (BCTs) on engagement [11], it appears vital to explore users’ psychological needs and preferences with regards to PA app engagement.

According to the person-based approach to intervention development, it is important to elicit and address the needs, perspectives, and experiences of potential users during the design process to develop apps that promote individual engagement [17]. To this aim, the use of mixed-methods research designs with a specific focus on qualitative methodologies (eg, focus groups, interview with open-ended questions, and think-aloud studies) has thus been recommended [17,18]. Previous research targeting different health-related behaviors in diverse populations has used existing commercial apps as stimuli to elicit users’ thoughts about factors that are expected to be important for engagement in real life [19-21]. Indeed, through the exploration and use of existing apps, potential users are prompted to reflect on their attitudes toward particular app features and how these might act as facilitators or barriers to uptake and adoption of the app.

### Objectives

The aim of this study was to guide the selection of design features to implement in PA apps for nonclinical, adult populations. Through a combination of think-aloud methodology and in-depth interview techniques, this study examined (1) what features potential users expect to be important for engagement with PA apps during first exposure to never-used, randomly allocated, and commercially available PA app and (2) what features are judged to be important for supporting engagement and satisfactory experiences after 2 weeks’ usage of the same PA app.

## Methods

### Study Design

This study used a longitudinal, single-arm design with a 2-week follow-up using think-aloud methodology and semistructured interview techniques. Since 74% of health apps are abandoned after their tenth use [12] and their usage tends to vary between a few times per week to twice per day [22], a 2-week period was considered to be sufficient to examine relevant changes in engagement with PA apps. Think aloud is a method that requires participants to verbalize their thoughts and impressions while performing a task [23]. The benefit of using think-aloud methodology is that it directly captures participants’ ongoing thought processes during a specific experience and, when applied to digital interventions, it allows researchers to observe and analyze users’ reactions to every element of the intervention [17]. In this study, participants were asked to verbalize their impressions, thoughts, and feelings while downloading and exploring a specific, never-used PA app randomly allocated from a pool of 3 apps identified by the researchers according to specific criteria (for more details about these criteria see the section “Mobile Apps”). Overall, 3 different apps were included as this allowed us to (1) expose participants to a greater number of intervention components (ie, BCTs) as shown in Table 1, and (2) obtain more valid conclusions and to be more confident in generalizing current results to other PA apps. Semistructured interviews were subsequently used to retrospectively investigate (1) statements made during the think-aloud task at baseline and (2) users’ thoughts and impressions after a 2-week period of *ad libitum* use of 1 of the 3 randomly allocated apps. The ethical permission for the study was granted before data collection by the ethics committee of the University of Milan-Bicocca.

**Table 1:** Behavior change techniques implemented in the selected physical activity (PA) apps, coded using the behavior change technique taxonomy (v1).

Behavior change techniques	Runtastic Running & Fitness Tracker	Endomondo - Running & Walking	Runkeeper - GPS Track Run Walk
1.1. Goal setting (behavior)	✔^a^	✔	✔
1.3. Goal setting (outcome)	—^b^	—	✔
1.4. Action planning	—	—	✔
1.9. Commitment	—	✔	—
2.2. Feedback on behavior	✔	✔	✔
2.3. Self-monitoring of behavior	✔	✔	✔
2.4. Self-monitoring outcomes of behavior	✔	✔	✔
2.7. Feedback on outcomes of behavior	✔	✔	✔
3.3. Social support	✔	✔	✔
4.1. Instruction on how to perform the behavior	✔	—	—
5.1. Information about health consequences	✔	—	—
5.4. Monitoring of emotional consequences	✔	—	✔
6.2. Social comparison	✔	✔	✔
9.1. Credible source	✔	—	—
10.5. Social reward	✔	✔	✔

^a^✔ indicates BCT is present.

^b^— indicates BCT is absent.

### Participants

Participants were recruited to represent a subsample of the Italian, adult, nonclinical population not meeting the PA guidelines and interested in using an app to increase PA. Participants were recruited in the Italian county of Lombardy and were eligible to take part in the study if they (1) were aged between 30 and 50 years (a more narrow age range was preferred to a wider one to prevent collecting data from a heterogeneous sample characterized by too varying motivations and subjective experiences); (2) did not have any pre-existing health conditions that would impede on the ability to engage in PA (eg, cardiovascular diseases, heart failure, and pulmonary conditions); (3) did not have a diagnosis of a clinical condition that would benefit from doing PA (eg, hypertension and diabetes) as clinical populations were likely to have different motivations compared with the intended users (ie, healthy adults); (4) reported PA behavior not in line with the recommended PA guidelines (ie, 150 min of moderate PA or 75 min of vigorous PA per week); (5) were willing to increase PA and interested in doing so using an app; and (6) owned an Android or iOS smartphone.

### Sampling

Participants were recruited through social media (eg, Facebook), snowball sampling methods (ie, each participant was asked to ﬁnd another participant), and posters placed in a large university campus. The aim of the study and the eligibility criteria were specified in the recruitment materials. Recruitment into the study was discontinued when no new insights and novel themes were identified in the interviews (ie, when theoretical saturation was achieved) [24].

### Procedure

Interested participants filled out a Web-based screening questionnaire to verify study eligibility. Eligible participants were contacted to schedule the baseline session and were asked to provide written informed consent before taking part in the study. Of them, 1 of 3 PA apps was randomly allocated to each participant before the baseline session. The baseline session included a pretask interview, a think-aloud task, and a semistructured interview (see Multimedia Appendix 1). After taking part in the baseline session, participants were asked to use the app for 2 weeks. No specific instructions were provided in terms of app use (eg, frequency or duration of use) to avoid influencing users’ engagement with and experiences of the app. Participants were, however, encouraged to use the app to increase their PA according to their own goals. Participants were contacted after 1 week to verify if they were using the app and to ask if they were willing to continue using the app for another week. The participants took part in a telephone interview 2 weeks after the baseline session using an interview schedule as guide (see Multimedia Appendix 1).

During the recruitment, each participant was given a unique code. A separate file in which codes were linked to personal identifiers (eg, name and contacts) was created, and any personal identifiers in the data file were removed and replaced with the codes. The de-identified data files and the file with personal identifiers were stored separately in secure locations, with access restricted to the study personnel.

### Measures

In the online screening questionnaire, participants were asked to report information about (1) age; (2) gender; (3) physical conditions that prevent participants from being physically active (eg, chronical back pain and acute cardiovascular disease); (4) physical conditions associated with a lack of PA behavior (eg, hypertension and obesity); (5) PA during leisure time, measured using the Global Physical Activity Questionnaire [25], with consequent assessment of whether PA was in line with the recommended guidelines; (6) intention to increase PA assessed through a 6-point Likert scale ranging from 1 (I do not intend to increase my PA level) to 6 (I intend to increase my PA level by the next month); (7) interest in increasing PA with the aid of a mobile app (yes vs no); and (8) ever use of a PA app. The screening questionnaire was hosted by Qualtrics software (Qualtrics, Provo, UT) [26].

### Mobile Apps

The PA apps identified for the study were *Runtastic Running & Fitness Tracker*, *Endomondo - Running & Walking*, and *Runkeeper - GPS Track Run Walk* (henceforth, *Runtastic*, *Endomondo*, and *Runkeeper*). The researchers have no association with the development or marketing of these apps. The inclusion criteria for the app selection were as follows: (1) their main goal was to increase PA among healthy adults, (2) they were freely available both on iTunes and Google Play, (3) they incorporated many BCTs (see Table 1 for a description of the BCTs incorporated in the selected apps, coded using behavior change technique taxonomy (v1) [27]) that are common among top-ranked publicly available PA apps [28], and (4) they had received high user ratings (ie, 4.5 stars), which is considered to be an indicator of positive user experiences with PA apps. Finally, it should be noted that all the selected apps contained advertisements and in-app purchases.

### Data Analysis

Quantitative data from the online screening questionnaire were analyzed using descriptive statistics with SPSS version 22 [29]. Qualitative data were audio-recorded and transcribed verbatim. Transcripts from the baseline and follow-up activities were analyzed using inductive thematic analysis [30]. Thematic analysis is characterized by 6 phases: (1) familiarizing with the data, (2) generating initial codes, (3) searching for themes, (4) reviewing themes, (5) defining and naming themes, and (6) producing the report. Baseline and follow-up transcripts were considered as 2 different datasets and were analyzed separately. Data and repeated patterns that were considered pertinent to the aims of the study were coded by the first author. New inductive codes were labeled as they were identified during the coding process and the results of the coding were iteratively discussed with 2 researchers (SN and SB). The next stage involved searching for themes; the first author reviewed the codes one-by-one, organizing the findings to combine different codes that focus on similar aspects. The ordered data were reviewed and revised in discussion with 2 researchers (SN and SB) and were subsequently organized into themes. Recruitment stopped when theoretical saturation was achieved (ie, no new themes were identified). Resolution of disagreements and agreement on the final themes was reached through discussion among all coauthors. After having defined and named themes, examples of relevant transcripts were selected to illustrate themes. Data were analyzed in their original language to preserve original meanings, although coding and themes were formulated in English only. Illustrative quotes were translated by the first and second authors.

## Results

### Descriptive Statistics

A total of 20 participants (55% male; mean age 39.8 years; SD 7.0) took part in the study. Overall, 35% of participants had made an attempt to increase their PA in the past 6 months and 35% had previously used a PA app to increase their PA. Among the 20 participants who completed the baseline session, 17 participants (17/20, 85%) completed the follow-up interview. In total, 2 participants were excluded as they did not use the app during the 2-week study period, and 1 participant could not be successfully recontacted. Table 2 presents information about app allocation, presence at follow-up, and self-reported app use (ie, frequency and duration of use). It should be noted that use is defined in terms of how frequently and for how long the apps were used to track PA; however, this might not necessarily be related to the overall duration of time spent doing PA during the study and it might not reflect the intensity of use (eg, the amount or depth of use per log-in).

### Thematic Analysis

In total, 6 themes were developed in relation to the first research question and were labeled: “fair and simple user experience,” “sense of autonomy and self-regulation of behavior,” “features that address users’ exercise motives,” “need for relatedness,” and “tailored support and action planning.” Overall, 1 subtheme was developed in relation to the second theme: “efficient and reliable monitoring and feedback of PA.” Overall, 3 subthemes were developed in relation to the fourth theme: “peer support,” “coaching support,” and “social comparison.”

A total of 5 themes were developed in relation to the second research question, 4 of which overlapped with themes from the baseline interviews. Hence, the labels were retained: “fair and simple user experience,” “sense of autonomy and self-regulation of behavior,” “features that address users’ exercise motives,” and “need for relatedness.” A new theme was developed, labeled: “proactive motivational features” (see Table 3 for a description of the themes and Multimedia Appendix 2 for additional extracts illustrating each theme).

#### A Fair and Simple User Experience

##### Baseline

Participants emphasized the importance of PA apps being able to promote a simple, easy, and fair interaction with its users. In particular, participants wanted the app to be simple to use, not cognitively demanding, or time consuming. Furthermore, participants expressed a strong dislike for obtrusive interactions such as inappropriate reminders or unexpected advertisements that redirect users to external websites:

It [user interface] should be simple and intuitive because this app is very complicated and I feel a bit lost.

P16 characterizing desired user interface during post-task interview

Participants were particularly annoyed when they were asked to manually enter information about themselves or their activity levels into the apps. Rather, they reported a preference for apps that automatically register their information:

I hate having to [manually] register everything I do!

P2 commenting on the user interface of the given app during think-aloud task

##### Follow-Up

After 2 weeks of *ad libitum* use, participants still maintained that simplicity and ease of use are essential prerequisites for app engagement. The perceived quality of the interactions participants had with their allocated app appeared to influence their overall satisfaction and affect their willingness to fully explore the app’s functionality:

I immediately understood how to use it and how to get it up and running.

P7 commenting on the user interface of the given app

It’s not so easy to find information or understand how it works. You need to spend some time playing around with it [...] it should be organised in an easier way.

P9 commenting on the user interface of the given app

**Table 2:** Information about app allocation to participants, presence at follow-up and self-reported usage of the app during physical activity session. Average use during the 2-week study was 4.3 times and average time duration per use was 73.6 min.

Participant	App	Follow-up^a^	Self-reported usage of the app during physical activity	
			Frequency of use during the 2-week study	Average time duration per use (min)
P1	Endomondo	✔	7 times	105
P2	Runtastic	✔	2 times	31
P3	Runkeeper	—	N/A^b^	N/A
P4	Endomondo	✔	6 times	65
P5	Runtastic	✔	6 times	45
P6	Runkeeper	✔	4 times	28
P7	Endomondo	✔	5 times	60
P8	Runtastic	✔	4 times	115
P9	Runkeeper	✔	7 times	75
P10	Endomondo	—	N/A	N/A
P11	Runtastic	✔	2 times	18
P12	Runkeeper	✔	2 times	30
P13	Endomondo	—	N/A	N/A
P14	Runkeeper	✔	1 time	50
P15	Endomondo	✔	5 times	35
P16	Runtastic	✔	7 times	N/A
P17	Runkeeper	✔	3 times	240
P18	Endomondo	✔	6 times	120
P19	Endomondo	✔	3 times	70
P20	Runkeeper	✔	3 times	90

^a^Presence at follow-up is denoted by ✔ while absence is denoted by —.

^b^N/A: not assessed.

**Table 3:** Summary of themes and subthemes identified in (1) the baseline interviews and (2) the follow-up interviews.

Theme and subthemes^a^	Description	Baseline	Follow-up
A fair and simple user experience	Features that enhance an easy, simple, and fair app interaction (ie, not cognitively demanding or time consuming)	✔^b^	✔
Features that promote autonomy and self-regulation of behavior	Features that support users’ autonomous behavior regulation toward personal goals (eg, self-monitoring, feedback, and goal setting)	✔	✔
*Efficient and reliable monitoring and feedback of PA*	Features that support the efficient monitoring of PA (physical activity) and related parameters (eg, calories and heart rate)	✔	✔
Features that address users’ exercise motives	Features that focus on health, fitness, and weight loss are expected to be motivating and engaging as they highlight the effects of PA on users’ individual exercise motives	✔	✔
Need for relatedness	Features that leverage the motivational component of social support and facilitate the interaction with human elements	✔	✔
*Peer support*	Features that leverage peer support to overcome laziness, increase commitment, and as a prompt to be more active	✔	—^c^
*Coaching support*	Coaching features that recommend what to do to achieve personal goals and facilitate the receipt of emotional support	✔	—
*Social comparison*	Features that allow users to share PA performance with others and to compete against one another	✔	✔
Tailored action planning	Features that help users to implement their exercise intentions and achieve goals through tailored plans	✔	—
Proactive motivational features	Features that prompt and stimulate users by sparking their interest and increasing motivation (eg, reminders and suggestions)	—	✔

^a^Subthemes are denoted by italics.

^b^✔ indicates theme is present.

^c^— indicates theme is absent.

#### Features That Promote a Sense of Autonomy and Self-Regulation of Behavior

##### Baseline

During baseline assessment, participants perceived monitoring, feedback, and goal-setting features as fundamental self-regulation components to build the entire app around, as these features were expected to support users’ autonomy and help regulate behavior toward their personal goals. Participants expected PA apps to include features that highlight their progress and failures; thus, clearly illustrating discrepancies between their current behavior and goals:

Looking at the percentage of achieved goals and getting a notification with relevant feedback saying: “You’re below average, above average, you are achieving your goal or not...” Well, it might be a way to remind me of my commitment and verify the percentage of accomplishments.

P15 commenting on existing and desired features during post-task interview; discrepancy between current behavior and goal and feedback on PA

##### Follow-Up

Participants maintained that self-regulation features such as monitoring, feedback, and goal setting had a positive effect on their engagement with the app. In particular, participants reported appreciating the possibility to monitor their progress over time and, consequently, regulate their behavior and effort:

I have to admit that it’s stimulating to be able to log all of my walks, how many calories I’ve burnt, the hydration I have lost, the average pace [...]. To me, [these features] are quite engaging, they make me more prone to open [the app] and log what I’ve done.

P1 commenting on existing features; self-monitoring of PA, feedback on PA, self-monitoring of outcome of PA, and feedback on outcome of PA

Participants also reported the need for autonomy and having an active role in the regulation of their behavior:

The goal shouldn’t be imposed [by the app] because in that case I won’t achieve it. The goal needs to come from inside - I don’t want to do X km because the app says so, but because I want to.

P8 commenting on existing features; goal setting

#### Subtheme: Efficient and Reliable Monitoring and Feedback of Physical Activity

##### Baseline

Participants expressed the desire for an app that efficiently monitors PA and related variables (eg, calories and heart rate). It was, therefore, considered important that the app could distinguish between specific types of PA and that it would provide tailored and reliable monitoring and classification of PA:

The app should be accurate, particularly the GPS! In order for me to use an app, it has to provide me with precise and not generic information, otherwise it is not worth using.

P8 characterizing desired features during post-task interview; self-monitoring of PA

##### Follow-Up

In line with the baseline interviews, monitoring features were considered to be important as they allow the collection of reliable information about PA and health-related parameters, hence enabling users to regulate their behavior:

It looks like there’s no possibility of assessing heart rate...in some apps, similar to this one, you can assess your blood pressure on your own with a blood-pressure monitor and verify your minimum and maximum blood pressure on a daily basis.

P1 characterizing desired features; self-monitoring of behavior and self-monitoring of outcome of PA

#### Features That Address Users’ Exercise Motives

##### Baseline

Participants reported an interest in apps that address their specific exercise motives. Health- and fitness-related participation motives were repeatedly reported as the main goals for being physically active. Specifically, participants expressed a preference for features that highlight fitness and weight loss. Hence, apps that allowed users to set calorie- and energy expenditure-related goals and monitor progress toward such goals were highly appreciated:

The fact that you can specify a training plan and, at the same time, control your heart rate and nutrition and figure out how these things interact...Well, in my opinion, these are the kinds of things that make an app engaging.

P1 characterizing existing and desired features during post-task interview; action planning, self-monitoring of PA, and nutrition

[The app] could, for example, be complemented by nutritional aspects...calories burnt, calorie intake.

P15 characterizing desired features during pretask interview; self-monitoring of PA and nutrition

##### Follow-Up

After 2 weeks of use, participants reaffirmed their positive attitudes toward features that take account of their personal motives for doing PA. In particular, nutrition suggestions, feedback about calories intake and consumption, and weight loss were thought to support engagement with PA apps:

If it was complemented and integrated with other things, such as calories, a diet, suggestions about nutrition – things that aren’t present in this app or that are paid – it would probably be more engaging. Otherwise people get bored after a while.

P15 characterizing desired features; feedback on outcome of PA and instruction on how to perform a behavior—nutrition

#### Need for Relatedness

##### Baseline

Participants highlighted the motivational aspect of *human elements*. According to most participants, PA apps should provide opportunities for social interactions, mostly conceived of as a way to obtain human, empathic support, thus fostering a higher level of commitment to one’s personal PA goal:

For me, it should be...I don’t know how to explain...a bit more human.

P14 characterizing desired features during pretask interview; social support—emotional

##### Follow-Up

After having used the app for 2 weeks, participants still highlighted the importance of app features that foster a sense of relatedness by creating a digitally enabled social environment that is supportive and nonjudgmental:

In my opinion, the app starts being enjoyable/entertaining when you have “group use.”

P16 characterizing desired features [social support—unspecified

However, different from the baseline interviews, the need for relatedness was mostly mentioned in relation to features that support social comparison. Hence, this constitutes the only subtheme.

#### Subtheme 1: Peer Support

##### Baseline

Opportunities for interacting with and doing PAs with peers were perceived as a way to boost one another’s motivation and to leverage social commitment through the organization of group activities. Indeed, users described peer support as a way to overcome laziness. They also expected that peer support would make exercise more enjoyable and that it would function as a prompt to be more active:

I believe that [doing PA with friends] is stimulating, it’s like when you go to the gym alone or with a friend, it’s the same idea, you can support each other.

P16 commenting on existing features during post-task interview; social support—emotional

#### Subtheme 2: Coaching Support

##### Baseline

Participants held positive attitudes toward the possibility of interacting with coaching features embedded into the app, as these were expected to provide practical exercise suggestions and to facilitate the receipt of emotional support:

The best way to attain a goal is to have a person that helps you...the app should be a substitute for a personal trainer.

P2 characterizing desired features during post-task interview; social support—practical

#### Subtheme 3: Social Comparison

##### Baseline

Most participants believed that sharing information about PA with one’s wider social network through an app is inappropriate and expressed the desire to avoid social comparison and maintain focus on their own goals:

I don’t like sharing...because I would come across as trying to brag, wouldn’t I?...when I do a specific thing it’s just for me, I don’t like to involve too many people.

P11 commenting on existing features during post-task interview; social comparison

However, some participants expressed a desire for more competitive features, contingent on competition being framed as a friendly way to support each other rather than competing against other users:

The opportunity to compete is great - everyone can set their goal and support each other!

P3 commenting on existing features during post-task interview; social comparison, goal setting, and social support—emotional

##### Follow-Up

Most participants had not shared personal results with other users when such features were available, as social comparison features were thought to expose users to others’ judgmental evaluations:

I’m not interested in sharing my runs and my results. I’m not a “social” person, so why would I start sharing the embarrassing results about my runs.

P2 commenting on existing features; social comparison

Although generally perceived as inappropriate, some participants mentioned that social incentives and challenges might make PA apps more engaging. Specifically, features that promote healthy competition were considered as a potential trigger to motivate users and make their exercise more enjoyable:

It would be interesting to get a group of friends together and say: “Let’s compete and see who’s training more, who’s burning more calories.” This would make [the app] more interesting.

P16 characterizing desired interaction with features; social comparison

#### Tailored Action Planning

##### Baseline

Features that help users to implement their PA intentions and achieve goals were judged to be important for engagement with PA apps. Participants expected PA apps to provide action plans to achieve individual goals:

They give you a training plan and you achieve your goals, that’s great!

P4 commenting on existing features during think-aloud task; action planning

When developing action plans, participants wanted the PA app to provide a step-by-step guide and provide users with suggestions and advice about how to exercise:

Something that gradually guides you towards your goals, step-by-step, perhaps also suggesting what kind of physical activity to do and providing advice.

P15 characterizing desired features during pretask interview; action planning and instruction about how to perform PA

#### Proactive Motivational Features

##### Follow-Up

Some participants expressed skepticism toward the app’s ability to boost motivation and to provide novel features that would spark their interest. According to these participants, the app was considered to be a functional tool rather than a trigger for initiating exercise:

I wasn’t particularly engaged, perhaps because it didn’t have additional functionalities to the other one that I had already used. This app didn’t stimulate me to search for information, look at my stats or to train more.

P8 commenting on the given app; general

Other participants who had enjoyed their app experience expressed positive attitudes toward proactive features such as reminders and suggestions that served as triggers both for doing PA and for supporting engagement with the app:

It provides suggestions about how much activity to do per week, how to increase it, etc. That’s what I liked a lot.

P1 commenting on existing features; instruction on how to perform PA and action planning

Moreover, some participants experienced a lack of proactive features and expressed their desire for a more interactive and prompting app:

I would have preferred it to remind me to exercise, like: “You are lazy, you should go out for a walk” [...] This app is too quiet, it’s not stimulating.

P11 characterizing desired features; prompts or cues

## Discussion

### Summary of Main Findings

This study examined what PA app features potential users expect to influence app engagement during first exposure to never-used, commercially available PA apps and after 2 weeks of app use. Participants’ thoughts and impressions were elicited through observing direct experiences of PA app use in the laboratory and through asking participants to report on their experiences of app use in their everyday lives to obtain ecologically valid insights. Thanks to the longitudinal study design, it was possible to compare what PA app features were considered important for engagement at different time points. A key advantage of the longitudinal qualitative study design was the ability to gain a more in-depth understanding of factors influencing users’ engagement with PA apps both initially and over a period.

At baseline and follow-up, participants expressed the desire for PA apps that are simple to use, intuitive, and not cognitively demanding. Our findings suggest that PA apps should be easy to use, avoiding confusing and inadequate user experiences, as these constitute barriers to engagement. This finding supports previous research, which highlights the importance of observing how users interact with apps in real time to assess their ease of use and how they fit within users’ everyday lives [18]. Participants in this study also highlighted the need for features that support the self-regulation of behavior to achieve personal exercise goals (eg goal setting, monitoring, and feedback), which supports previous findings [19]. The positive effect of personal goal attainment on individual well-being has been largely recognized [31] and the goal regulation process has been specifically described by Carver and Scheier [32] as a discrepancy-reducing feedback loop in which individuals are motivated to self-regulate their actions according to feedback to achieve a specific goal. Our findings suggest that the availability of self-regulation features might allow users to self-organize their experiences and behavior and hence, as proposed by self-determination theory [33], foster experiences of autonomy. Our findings, corroborated with results from recent reviews and qualitative studies, indicate that app features that support self-regulation are expected to increase engagement [11,16], are appreciated by users [34], and are associated with PA intervention effectiveness [4,35]. This suggests that self-regulation features should be considered as core components of PA apps.

Addressing users’ PA participation motives emerged as a further important aspect of engagement with PA apps. In line with previous research [13], most participants preferred features that focus on fitness, nutrition, and weight loss as these were the main reasons for engaging in PA. For instance, some participants were strictly interested in suggestions about how to lose weight and wanted to track relevant parameters (eg, weight loss and calorie intake). Although intrinsic exercise motives, relative to more controlled ones (eg, body-related motives), have been shown to be positively associated with PA [36], some forms of less autonomous regulation (eg, identification with the outcomes of PA) are expected to regulate short-term behavior [37] and constitute an important motivational component of exercise [38]. Hence, app features that address users’ exercise motives might be leveraged as a trigger for engagement with PA apps as well as for boosting motivation in the early stages of behavior change.

Users expressed varying types and levels of needs relating to the connectedness with other users. Some participants believed that opportunities for connecting with peers might help to develop a climate of social commitment that increases motivation and makes exercising a more enjoyable activity. However, the subtheme labeled *peer support* was not identified during the follow-up interviews. This might be because of the fact that participants, in spite of their initial inclination to seek support from peers, were unwilling to connect with strangers or because they were worried by social comparison.

The motivational aspect of human support was also mirrored by users’ preferences for coaching features directly embedded into PA apps. These results support those from a focus group study about preferred PA app features [19], which found that coaching features were thought to be as an advantage in PA apps. Moreover, these findings are consistent with previous research suggesting that the desire to continue working with a digital behavior change intervention is higher when supported by the presence of human relational skills (eg, empathy and social dialog) designed into a computer interface, as this fosters the therapeutic *working alliance* [39]. Moreover, findings from a scoping review of Web-based interventions highlighted the potential for supportive virtual coaches to counteract low adherence in digital behavior change interventions [40]. It is worth noting that this subtheme was not identified during the follow-up interviews. A plausible explanation for this is that coaching features, which were mentioned as desirable by participants during the baseline interviews, were completely lacking from the selected PA apps.

Consistent with previous research into different behaviors [20,34], potential users of PA apps were reluctant to share PA information with their social networks. In our study, most participants thought that social comparison features would hinder their motivation to exercise because of exposure to others’ negative judgments. However, some participants wanted to take part in social challenges, contingent on these being *friendly*, thus facilitating the receipt of mutual social support.

An additional theme labeled *tailored action planning* emerged only during the baseline interviews and relates to the role of the app in supporting users to implement their intentions and to achieve their goals. Users expressed their desire to be provided with tailored PA plans and suggestions based on their goals and current progress. This highlights the crucial role of action planning features in PA apps to facilitate goal attainment [41]. This theme was not identified as important during the follow-up interviews. A possible explanation is that action planning is a volitional rather than a motivational variable [41]. Hence, although action planning may help individuals to implement their intentions, it does not motivate and nudge PA app users to engage with the digital behavior change intervention itself over time.

After having used the app for 2 weeks, participants expressed the desire for PA apps with proactive features that boost their motivation. Participants who enjoyed their allocated app mentioned their liking of proactive features (eg, suggestions and reminders), and these were also mentioned as a point for improvement by participants who were not satisfied with their allocated apps. Hence, the development of a proactive app that is able to tailor the intervention content and timing according to individual differences is hypothesized to be a key element for supporting engagement with PA apps. These results are in line with recent advances in intervention design aimed at the development of adaptive interventions (eg, just-in-time adaptive interventions) that integrate behavioral, psychological, and contextual information to deliver more tailored and potentially effective strategies to increase PA [42,43]. Proactive features may actively support users’ self-regulation skills, reducing users’ responsibility for the behavior change process. Although this theme was not identified during the baseline interviews, it represented a key issue in the follow-up interviews when participants were prompted to reflect on their actual app use. This finding provides support for the argument that sustained engagement with PA apps cannot simply rely on users’ motivation. Instead, motivation to continue engaging might be the result of moment-to-moment engagement that is triggered by motivational and proactive app features.

### Implications

Thanks to the longitudinal design, this study facilitated the development of a comprehensive picture of potential users’ needs and preferences regarding PA apps, with clear implications for the development of future, or modification of existing PA apps for physically inactive adults.

The findings that potential users are interested in self-regulatory features supported current design practice in PA apps; however, some improvements can be introduced. First, users should be able to monitor and assess the PA they perform in a more reliable and efficient way. Second, features that enable users to draw their attention to how their PA progresses and to any discrepancy between PA behavior and goals may help to guide their self-regulation and efforts.

As potential users are likely to practice PA to improve fitness and lose weight, PA apps should (1) deliver suggestions about what exercises to do and how to exercise to achieve specific health-related goals, (2) provide nutritional advice and opportunities for monitoring food intake, (3) monitor and provide feedback on health-related progress (eg, weight loss, waistline reduction, and blood pressure), and (4) permit users to estimate the daily and weekly difference between calorie intake and consumption.

While interacting with PA apps, simplicity and usability influenced users’ perception of the app. Indeed, simple apps were seen as more easy to use and, thus, more immediate and useful. In particular, users expected that apps should not be cognitive demanding or time consuming. Therefore, developers should focus on designing PA apps that assure immediate and efficient use, avoiding poor user experiences that constitute a barrier to engagement and behavior change. To this end, design and implementation phases should be characterized by a constant and iterative evaluation of the user experience in real-world settings.

Further findings suggested that the design of PA apps may be improved by providing opportunities to connect users to one another and to easily arrange for social PA events. As pointed out, there may also be merit in designing PA apps that interact with users in the same way as a personal coach. Hence, developers may consider the opportunity to characterize PA apps by the presence of human relational skills or components (eg, virtual coaching, empathy, and social dialog).

Finally, proactive features (human support, tailored PA plans and suggestions, and context aware prompts) constitute one of the main gaps of commercial PA apps. This is a crucial point because, as emerged in this study, the development of a proactive app that is able to tailor the intervention content according to individual differences and diverse circumstances is hypothesized to be a key element for supporting effective and sustained engagement with digital interventions. To this end, a close collaboration between behavioral and computer scientists is required for developing machine learning techniques that can help understanding, predicting, and meaningfully addressing users’ needs.

### Limitations

This study also has a few limitations. First, participants’ heterogeneous levels of digital literacy might have influenced the quality of the experience with the apps. For example, it is possible that some participants had difficulties with their allocated apps because of low digital literacy. We tried to minimize such limitations as far as possible by only including top-ranked apps, thus preventing participants from interacting with low-quality user interfaces. Second, we asked participants to use the app for 2 weeks to preserve the ecological validity of the study findings but did not specify amount and frequency of use. However, it is possible that the accuracy and depth of participants’ answers in the follow-up interviews was influenced by the amount and frequency of app use during the 2 weeks. Moreover, 3 freely available apps were chosen for this study as stimuli to elicit participants’ needs and preferences. Although the choice of these apps was based on criteria aiming to provide participants with high-quality apps, it is possible that a greater number of apps might have elicited different and even more generalizable insights. However, we believe that our findings may generalize to other commercial apps as the selected ones were characterized by most of the BCTs generally implemented in PA apps [28]. Finally, the 2-week longitudinal study design was aimed at understanding what and why particular app features sustained engagement over time. The second time point was defined on the basis of statistics suggesting that 74% of health apps are abandoned after their tenth use [12] and their usage mainly varies from a few times each week to 2 times each day [22] and, consequently, assuming that abandonment may occur around the end of the second week of use. Our results confirmed previous statistics [22]; however, none of the participants used the app for more than 7 times. Therefore, findings from this study cannot answer the question what and why design features influence engagement with PA apps over a longer period.

### Conclusions

Our findings suggest that designers of PA apps may benefit from taking into account users’ exercise motives (ie, fitness and weight loss) and supporting users with self-regulation features such as monitoring, feedback, and goal setting. As participants expressed a desire for features that foster the sense of relatedness, features that leverage the motivational aspects of relational and emphatic support in the behavior change process should be considered in the design of PA apps. Similarly, preferences for tailored action planning and proactive features drew attention to the need for the active role of the app as a complement to other features that support users’ self-regulation skills.

## Appendix

Multimedia Appendix 1 Think-aloud and semistructured interview protocol for baseline and follow-up.

Multimedia Appendix 2 Additional excerpts from the think-aloud and interview sessions illustrating each theme.

## Abbreviations

BCT: behavior change technique
PA: physical activity
