# Supplementary material for: Exploring Users’ Experiences of the Uptake and Adoption of Physical Activity Apps: Longitudinal Qualitative Study
Source: JMIR Mhealth Uhealth. 2019 Feb 8;7(2):e11636. doi: 10.2196/11636 (PMC6384536; doi:10.2196/11636)
Supplement: Multimedia Appendix 1 [file mhealth_v7i2e11636_app1.pdf]

MULTIMEDIA APPENDIX 1

<u>Baseline</u>

Think aloud activity

Pre-task interview

- Can you tell me about an app that you are using regularly?
- Have you ever used a health or fitness app? Can you tell me about it?
- What do you think a physical activity app should provide or do?
- What is your relationship with physical activity and sport? What do you think about it?

Verbal instructions

"During this session, you will be given one smartphone-based task to complete. I would like to emphasise that this is not a test; I am interested in the task themselves, not your performance. I would like you to complete the task whilst "thinking aloud". This means that I would like you to complete the tasks, and while you do so, try to say everything that goes through your mind. I would like you to pretend that you are at home and try to forget that I am here.

Thinking aloud usually feels a bit strange at first, as it is an unusual task. Don't worry about it, most people find it a bit unnatural at first, but quickly get used to it! We will start off with a practice task to make sure that you feel comfortable. I would like you to change the ring tone on your smartphone whilst trying to say everything that goes through your mind."

Task

"Imagine that you have selected a physical activity (randomly assigned) app that you would like to try. Please download it, complete the baseline questions and explore the app whilst thinking aloud. Feel free to explore the app as you prefer but pretend that you are really checking that the app fits with you interests and expectations."

Post session interview

- Ask about the participant's ability to download and install each app
- I noticed that you mentioned that you thought that [...] was ... Can you tell me a bit more about that?
- I noticed that you made a comment about [...]. Can you elaborate on that?
- How do you understand the term "engagement" in the context of apps?
- Do you think that this app was engaging? Why/why not?
- Do you think that you would find the app/those particular features engaging longer term? Why/why not?

- You mentioned that you thought that [...] was ... How do you think that feature would fit into your daily life?
- How important is it for you to be able to relate to the app's content?
- How do you think engaging with the app would help you to increase your physical activity?
- How do you think that [...] would help you to increase your physical activity?

<u>Follow-up (after 2-week use)</u>

Semi-structured interview (audio recorded phone call)

- Ask participant to confirm which app they have tried
- Ask participant to:
- Start by giving overall opinion of the app
- Depending on amount of detail provided in overview, ask participant to:
- Expand on any points raised in overview
- Comment on likes/dislikes
- Comment on specific app functionalities (linked with BCTs)
- Characterise the app in term of engagement. Get information about:
- Enjoyment (like/dislike). Ask for a justification.
- Interest (interesting/not interesting). Ask for a justification.
- Attention (superficial/deep experience). Ask for a justification.
- Frequency of use (how many times the app has been used)
- Intensity of use (how much time last each session more or less)
- Discuss how appropriate and relevant the app was for their personal intention to increase physical activity
- Discuss if the specific type of proposed PA fits with their interests or not
- Discuss types of physical activity which should be promoted, including intensity, frequency, type of activity and with relevance to current physical activity guidelines (i.e. 150 minutes of moderate to vigorous PA and 2 sessions of strength/resistance based exercises per week) and how apps could promote these types of physical activity (if at all).
- Discuss how (if at all) the apps could be improved. If so, what adaptations/functions should be addressed.
