# Supplementary material for: Exploring Users’ Experiences of the Uptake and Adoption of Physical Activity Apps: Longitudinal Qualitative Study
Source: JMIR Mhealth Uhealth. 2019 Feb 8;7(2):e11636. doi: 10.2196/11636 (PMC6384536; doi:10.2196/11636)
Supplement: Multimedia Appendix 2 [file mhealth_v7i2e11636_app2.docx]

**MULTIMEDIAL APPENDIX 2**

| **A FAIR AND SIMPLE USER EXPERIENCE** |
| --- |
| ***Baseline*** |
| *“It [user interface] should be simple, without too many things that make life difficult” P11 characterising desired user interface during post-task interview* |
| *“If I need an instruction manual and one week to understand how it works, I will give up” P1 characterising desired user interface during post-task interview* |
| *“If I have to spend hours and hours looking for information, I will give up” P14 characterising desired user interface during post-task interview* |
| *“For someone like me, who’s not used to have the smartphone next to me at all times and play around with it, it becomes demanding after a while and you have to stop what you are doing and focus on registering everything” P1 commenting on the user interface of the given app during post-task interview* |
| ***Follow-up*** |
| *“I liked it because I it was very easy to use” P6 commenting on the user interface of the given app* |
| *“I actually managed to use it once but the other times I couldn’t understand how to get it started.” P13 commenting on the user interface of the given app* |
| *“There are annoying advertisements that pop up every now and then because this is the free version of the app” P15 commenting on the user interface of the given app* |
|  |
| **FEATURES THAT PROMOTE AUTONOMY AND SELF-REGULATION OF BEHAVIOUR** |
| ***Baseline*** |
| *“…you have to achieve tangible improvements and be able to log them in order to be conscious of your improvements” P6 commenting on existing features [goal setting, self-monitoring of PA] during post-task interview* |
| *“What motivates you after a run is looking at what you have done. If you do this on a daily or regular basis, you can say: last week I did a certain amount of kilometres in a specific time, now I have improved it or not; or it is 15 days since I did any physical activity. You can monitor and regulate yourself.” P3 commenting on existing [feedback on PA, self-monitoring of PA] during post-task interview* |
| *“…accuracy in terms of the physical activity that you have done! Some of [the apps] erroneously display walking routes that I haven’t done… [I want] something with better capacity.” P16 characterising desired features [feedback on PA, self-monitoring of PA] during pre-task interview* |
| ***Follow-up*** |
| *“The most interesting thing that a physical activity app should do is that it should inform you about how many calories you’ve burnt, how many kilometres you have done and finally it should use GPS data to inform you about your route.” P2 commenting on existing features [self-monitoring of PA, feedback on PA, feedback on outcome of PA]* |
| *“Well, I think it is the most prompting thing. I mean, it is all here in black and white… in my opinion, it’s more stimulating.” P4 commenting on existing features [feedback on PA]* |
| *“It’s been enjoyable and interesting realising how much I walked during the four days I spent abroad” P9 commenting on existing features [feedback on PA]* |
| *“First of all, [the app] allows me to verify what I have done each week and if I’m improving or worsening my performance over time.” P8 commenting on existing features [feedback on PA]* |
| *“It allows you to rank your performance and provides you with feedback about your improvement or decline.” P18 commenting on existing features [feedback on PA]* |
| *“I liked it because, as I told you, this diary records what you do and it’s nice realising how you change over time” P11 commenting on existing features [feedback on PA]* |
|  |
| **FEATURES THAT ADDRESS USERS’ EXERCISE MOTIVES** |
| ***Baseline*** |
| *“It depends on the results you want to obtain and on the goal you set. In my case, for example, the goal is to lose weight.” P6 commenting on existing features [goal setting outcome] during post-task interview* |
| *“Losing weight might be another goal, related both to nutrition and exercise performance.” P7* |
| *“It would be great to have stats about your weight in order to see how it changes over time.” P18 characterising desired features [feedback on outcome of PA] during post-task interview* |
| ***Follow-up*** |
| *“It displays calories burnt based on time spent exercising, that we women love so much because it makes us think that we look leaner.” P12 commenting on existing features [feedback on outcome of PA]* |
| *“I realised that I could connect it to a nutrition app with regards to what to eat and that’s what I liked.” P4 commenting on existing features [connecting to nutrition app]* |
| *“Certainly, the part about nutrition might be interesting, seeing all your data and your nutritional habits and understanding how the app can help you to achieve a healthier and more balanced diet.” P9 characterising desired features [self-monitoring of nutrition, feedback on nutrition, instruction on how to perform a behaviour - nutrition]* |
|  |
| **NEED FOR RELATEDNESS** |
| ***Baseline*** |
| *“Your friends can... wow! support you” P3 commenting on existing features [social support - emotional] during think aloud task* |
| *“In some commercial apps you can chat with other people who answer in real time, in case of doubt or if you have any questions.” P13 characterising desired features [social support - unspecified] during think aloud task* |
| *“If I’m motivated and I receive a message saying: “Oh, come on! You decided to do it, let’s do it!”, it would prompt me [to exercise]” P11 characterising desired features [social support - emotional] during post-task interview* |
| ***Peer support*** |
| *“I’m used to doing these things on my own because I know that I’m not consistent [in my exercise patterns]… maybe having a group may help to be more consistent.” P11 commenting on existing features [social support - unspecified] during post-task interview* |
| *“Maybe I can ask a friend to run together and, once the half-hour run is finished, we can recover while chatting.” P1 characterising desired features [social support - unspecified] during post-task interview* |
| *“Or maybe with someone else who usually runs; that way you can say: “let’s have the same [exercise] plan and train together”. This is something that works!” P7 characterising desired features [social support - unspecified] during post-task interview* |
| ***Coaching support*** |
| *“I think the app should work as a personal trainer at the gym.” P14 characterising desired features [social support - practical] during post-task interview* |
| *“Something that progressively monitors you, like if I had a personal trainer next to me. In order to be really useful, the app should be of this kind.” P20 characterising desired features [social support - practical] during post task interview* |
| ***Social comparison*** |
| *“If I had a goal, like accomplishing a workout or running for X days, I would not be thrilled to share what I do with others.” P15 commenting on existing features [social comparison] during post-task interview* |
| *“For me [the app] shouldn’t have too many links to social networks because it has to be a private thing.” P6 commenting on existing features [social comparison, social support - unspecified] during pre-task interview* |
| *“I would be annoyed by sharing information about what I am doing with others. Honestly, it’s my business and the comparison is just with myself.” P1 commenting on existing features [social comparison] during post-task interview* |
| *“I would consider competition from an ironic perspective, I’m no longer at the age where I’d like to compete against anybody. In terms of groups, it would be better to find someone with the same motivations as you.” P11 commenting on existing features [social comparison] during post-task interview* |
| *“I’m very competitive, so I’ll only take part in competitions when I’m in my top condition.” P2 commenting on existing features [social comparison] during post-task interview* |
| *“Ranking with friends… no, I don’t want to compete right now, I’m terrible.” P3 commenting on existing features [social comparison] during think aloud task* |
| ***Follow-up*** |
| ***Social comparison*** |
| *“I would remove the connection with friends and social networks because it’s not necessary to me. Indeed, I believe they are personal activities that don’t have to be shared.” P6 commenting on existing features [social comparison]* |
| *“Maybe it’s possible to compete against other unknown users in order to increase one’s own performance.” P8 characterising desired features [social comparison]* |
|  |
| **TAILORED ACTION PLANNING** |
| ***Baseline*** |
| *“Here, this is a nice thing: make a plan based on your goals.” P1 commenting on existing features [action planning] during think aloud task* |
| *“It’s like having a personal trainer… if someone wants to train for a specific goal.” P16 commenting on existing features [action planning, social support - practical] during post task interview* |
| *“I would just like to set my goal, for instance, marathon… marathon in six months… telling [the app] what I want to achieve and within what time frame and that’s it… based on that it should give me a couple of plans to achieve my goals.” P17 characterizing desired features [action planning] during post-task interview* |
| *“At least they [training plans] provide you with an idea about what to do because I’m a beginner and have no idea about how to start training. The plans properly explain to you what to do instead.” P4 commenting on existing features [action planning] during post-task interview* |
|  |
| **PROACTIVE MOTIVATIONAL FEATURES** |
| ***Follow-up*** |
| *“You need a lot of motivation to use the app and go out for a run. Even the right the app can’t do so much.” P2 commenting on the given app [general]* |
| *“When I went out for a run, it was thanks to my own motivation, not because of the app honestly.” P14 commenting on the given app [general]* |
| *“I can say that the app in itself, no, it didn’t stimulate me enough to change my level of physical activity. I’m pretty sure.” P17 commenting on the given app [general]* |
| *“I mean… [the app] has not any added value, it provides nothing more [than my own motivation].” P17 commenting on the given app* |
| *“There is some advice that help you to gradually increase your confidence in doing physical movements.” P12 commenting on existing features [instruction on how to perform PA]* |
| *“I appreciated the alert… the fact that it tells you that today is Wednesday and you have to train.” P14 commenting on existing features [prompts/cues]* |
| *“The other positive aspect is the alerts that are not annoying but, rather, stimulate use of the app… it’s the right frequency of alerts.” P15 commenting on existing features [prompts/cues]* |
| *“To get a 5-star engagement rating would require a person that wakes you up and says: “Let’s go running with me!” P2 characterizing desired features [social support – unspecified, prompts/cues]* |
